# Data sharing between home care professionals: a feasibility study using the RAI Home Care instrument

**DOI:** 10.1186/1471-2318-14-81

**Published:** 2014-06-30

**Authors:** Dawn M Guthrie, Robyn Pitman, Paula C Fletcher, John P Hirdes, Paul Stolee, Jeffrey W Poss, Alexandra Papaioannou, Katherine Berg, Helen Janzen Ezekiel

**Affiliations:** 1 Department of Kinesiology and Physical Education, Wilfrid Laurier University, 75 University Ave. W, Waterloo, ON N2L 3C5, Canada; 2 Department of Family Relations and Applied Nutrition, University of Guelph, 50 Stone Rd. E, Guelph, ON N1G 2W1, Canada; 3 Ontario Home Care Research and Knowledge Exchange Chair, School of Public Health and Health Systems, University of Waterloo, 200 University Ave. W, Waterloo, ON N2L 3G1, Canada; 4 Department of Medicine, Director, Division of Geriatric Medicine, McMaster University, Chedoke Hospital, Wilcox Building, Sanatorium Road, Hamilton, ON L9C 1C4, Canada; 5 Department of Physical Therapy, University of Toronto, 160-500 University Ave., 8th Floor, Toronto, ON M5G 1V7, Canada; 6 Waterloo Wellington Community Care Access Centre, 450 Speedvale Avenue West, Suite 201, Guelph, ON N1H 7G7, Canada

**Keywords:** Home care, Rehabilitation, InterRAI, Standardized assessment, Information sharing

## Abstract

**Background:**

Across Ontario, home care professionals collect standardized information on each client using the Resident Assessment for Home Care (RAI-HC). However, this information is not consistently shared with those professionals who provide services in the client’s home. In this pilot study, we examined the feasibility of sharing data, from the RAI-HC, between care coordinators and service providers.

**Methods:**

All participants were involved in a one-day training session on the RAI-HC. The care coordinators shared specific outputs from the RAI-HC, including the embedded health index scales, with their contracted physiotherapy and occupational therapy service providers. Two focus groups were held, one with care coordinators (n = 4) and one with contracted service providers (n = 6). They were asked for their opinions on the positive aspects of the project and areas for improvement.

**Results:**

The focus groups revealed a number of positive outcomes related to the project including the use of a falls prevention brochure and an increased level of communication between professionals. The participants also cited multiple areas for improvement related to data sharing (e.g., time constraints, data being sent in a timely fashion) and to their standard practices in the community (e.g., busy workloads, difficulties in data sharing, duplication of assessments between professionals).

**Conclusions:**

Home care professionals were able to share select pieces of information generated from the RAI-HC system and this project enhanced the level of communication between the two groups of professionals. However, a single information session was not adequate training for the rehabilitation professionals, who do not use the RAI-HC as part of normal practice. Better education, ongoing support and timely access to the RAI-HC data are some ways to improve the usefulness of this information for busy home care providers.

## Background

Home care is an increasingly important sector of the health care system. In Canada, approximately $3.4 billion was allocated in the public budget for home care, which represents roughly 4.3% of total Canadian health care expenditures [1]. Ontario alone spends roughly $1.9 billion on home and community support services annually [2]. In Ontario, home care services are provided by Community Care Access Centres (CCACs) which represent a single point of entry for home-based services such as nursing, physiotherapy, occupational therapy, dietetics, social work and personal support, as well as admission to long-term care facilities. There are 14 CCACs across the province that employ care coordinators (previously referred to as ‘case managers’ in Ontario, which is the term used frequently by our focus group participants) who assess client needs, set goals with client/caregivers, determine eligibility, oversee service delivery and provide care coordination and system navigation. The in-home services are provided by external organizations that enter into a contractual agreement with the CCAC. Each year, CCACs serve roughly 600,000 Ontarians, over half of whom are older adults (aged 65+) [2].

In 2002, the Ontario government mandated the use of a standardized assessment tool, the Resident Assessment Instrument for Home Care (RAI-HC) [3], for all adult, non-palliative long-stay clients. The RAI-HC was developed by interRAI (http://www.interrai.org), a non-profit research consortium [4,5], and has documented reliability and validity [3,6-8]. A key benefit of the RAI-HC is that it captures data at the level of the individual client in a way that is consistent and standardized, which has been cited as a key facilitator for using health information in home care [9]. Implementation and ongoing use of this system represents a substantial investment, yet some early evidence suggests that this information is not currently being utilized in Ontario to its fullest potential [10]. Anecdotal information from the Waterloo-Wellington CCAC confirmed that there were inconsistencies within their organization regarding the sharing of the data with their contracted service providers.

A lack of coordinated information exchange between providers can lead to a duplication of efforts, increased assessment burden, increased frustration for the providers and care recipients [11], a disruption in care provision [12] and potentially poor client outcomes if potential clinical issues are not recognized by all care providers. Although it is recognized that communication between professionals is vital, there is limited understanding regarding how professionals in the home care sector share information and work together to address complex needs among older adults [13].

This project involved a process of data sharing between the Waterloo-Wellington CCAC (located in southwestern Ontario) and their contracted rehabilitation providers for older adults who had experienced a recent fall. We chose this clinical area since falls have a prevalence of roughly 25% in Ontario home care clients [14,15], falls prevention was a targeted area of interest to this CCAC and falls cost the Canadian health care system roughly $3.0 billion each year [16]. Environmental hazards in the home represent an important cause of falls [17] and roughly 50% of falls that lead to hospitalization occur in the home [18], reinforcing the need for home-based prevention strategies.

Falls are a complex issue among older adults and there is some evidence emerging that the most effective community-based strategies are multidisciplinary and target multiple risk factors [19]. The literature on falls prevention provides clear guidance on the qualities of an effective prevention strategy. As such, we did not develop a new falls prevention strategy, but rather focused on education and sharing of the RAI-HC data.

The overall goal of this one-year pilot was to collaborate with the CCAC, and their rehabilitation therapy providers, to develop and implement a data sharing protocol that represented a new way of communicating. We set out to assess whether this type of intervention could be feasible within the everyday practice of providing home care and to determine, from the perspective of the care coordinators and the contracted therapy providers, what benefits and challenges arose while taking part in this pilot project.

## Methods

Care coordinators completed client re-assessments, as part of usual practice, using the RAI-HC and the associated Clinical Assessment Protocols (CAPs). The RAI-HC contains roughly 300 items that cover domain areas such as functional ability, cognition, pain, mood, communication and service use [20]. Items within the assessment are linked to a series of 30 different CAPs that flag areas of risk for the client (e.g., falls, long-term care admission, cognitive disorders) and provide detailed written documentation to assist care coordinators in developing a comprehensive service plan. The software system creates a summary report that highlights which of these areas were identified for a given client.

Long-stay home care clients (aged 55+) being re-assessed between July 1, 2009 and April 30, 2010 were eligible to be in the study. A fall was defined as “any unintentional change in position where the person ends up on the floor, ground, or other lower level” within the previous 90 days of the assessment, as outlined on the RAI-HC [20]. A ‘new fall’ was one that occurred within the last 90 days for clients who did not experience a fall on their previous RAI-HC assessment. For these clients, the care coordinator faxed a referral package to the contracted therapy provider that included a summary of the information from the RAI-HC (as detailed below). The therapists also completed other assessments as part of normal clinical practice (e.g., the Berg Balance Scale [21,22] and the Timed Up and Go [23]). Clients who did not have a new fall did not receive rehabilitation services but did receive other home care services as determined by the care coordinator. All clients assessed with the RAI-HC during this time frame also received a brochure focusing on falls prevention and some education from the care coordinator about ways to avoid falls in the home. The brochure was a one-page (double-sided) document which was developed by several members of our team (e.g., Drs. Hirdes, Fletcher, Berg) as part of a previous study. The actual discussion about the brochure and falls prevention was not standardized, but was left to the discretion of the care coordinator. Current guidelines suggest that client education should be part of a multidisciplinary intervention [24].

### Education on the RAI-HC

All new care coordinators, within the participating CCAC, receive a very detailed four-day training as part of standard practice. New learners then spend one day with an experienced RAI user after their in-classroom training and return within 6 weeks for a RAI-HC Reflection/Refresh day to review coding and the various outputs from the RAI-HC assessment. For example, once entered electronically, several health index scales can be generated directly from the RAI-HC data (e.g., Cognitive Performance Scale [25], the Depression Rating Scale [26] and MAPLe score [27], which assists care coordinators with prioritizing clients for long-term care placement). Based on earlier work [28], and informal discussions with the CCAC, it was evident that the contracted therapy providers typically did not receive this level of training and did not have a detailed understanding of how to use the RAI-HC and its applications.

To ensure a consistent level of knowledge about the RAI-HC and its applications, a one-day education session was held for the care coordinators (n = 12) and service providers (n = 16). The session was led by a Master’s trained clinical educator with many years of experience in providing instruction on these instruments.

### Data sharing

Prior to this study, the RAI-HC assessment itself was not shared with the therapy providers as it was generally felt to be too cumbersome (roughly 12–14 pages) and the service providers typically did not have the level of expertise to fully interpret and utilize this information. As such, the care coordinators spent a substantial amount of time pulling relevant information from the RAI-HC, on a client by client basis, to create summary notes to be shared with the service providers. The research team worked closely with a Clinical Advisory Group throughout the entire study. This group included the study principal investigator (DG), the study research coordinator (RP), the Senior Director of client services for the CCAC, the Manager of Client Services for the CCAC (HJE), four care coordinators and senior level representatives from each of the four participating therapy providers. This group determined what information would be shared between the care coordinators and the participating service providers and decided on the following: the client information summary (that is normally shared by the CCAC with all referrals), the Personal Health Profile (a 2-page summary of the RAI-HC), the care coordinator and consultation notes, a summary of the relevant CAPs identified for the client, the MAPLe score and the falls checklist. This checklist was a one-page document, developed for the project, which summarized the risk factors for falling that were relevant for the client and the “next steps” to address these including further assessment, referrals and other interventions.

### Focus groups

At the end of the project, all participating care coordinators and service providers (total of 28 individuals) were invited by the study research coordinator to take part in a focus group and ultimately, two separate focus groups were held, one with care coordinators (n = 4) and one with service providers (n = 6). Focus groups were chosen since the participants logically fell into two clinical groups and the topic was not something that was sensitive in nature. These sessions were led by a qualitative researcher with 20+ years of experience who was not a part of the research team. Both sessions were held at Wilfrid Laurier University and lasted approximately 2 hours. The focus groups were semi-structured and asked questions related to the most positive aspects and challenges of being involved in the project, the education session, the usefulness of the falls CAP, whether they would recommend continuing to share the RAI-HC data (after the study was completed), what were the benefits and challenges with sharing the RAI-HC data and what advice they would have for the research team if they were to implement a similar project in the future. Informed consent was obtained from each participant prior to participation in the focus groups. Both sessions were audio-recorded and subsequently transcribed verbatim. All digital audio files and transcripts were saved on a password-protected computer accessible only to the study research coordinator. Names were not used in the transcripts and participants were identified with a unique identifier. The study protocol was approved by the Wilfrid Laurier University Research Ethics Board (reference #2336) and all procedures were in compliance with the Helsinki Declaration.

### Credibility of the data

Utilizing data triangulation (i.e., multiple data collection methods: information from the two focus group sessions, the background information on the participants and field notes gathered by the interviewer) and investigator triangulation (i.e., multiple researchers for analyses, background of focus group leader) strengthened the credibility of the data [29].

### Data analysis

A critical content analysis was conducted for all sources of data collected. Two members of the research team analyzed the data independently and then met to discuss emerging themes within the data. Neither of these individuals were involved in the actual focus groups. This paper will specifically focus on two main themes: (1) Successes of the Project; and (2) Improving the Process. These themes were chosen since roughly 90% of participants supported each theme. The quotations provided are words said verbatim by the participants in order that the “voice” of each of the participants was conveyed.

## Results

In order to protect the privacy and anonymity of the focus group participants, limited background information will be presented. Among the 10 participants, 9 were female, and among the 6 service providers, 4 were physiotherapists and 2 were occupational therapists.

### Theme 1: Successes of the project

Participants from both focus groups reported that there were many positive or successful elements associated with this pilot project. The care coordinators primarily talked about the beneficial effects of the falls brochure. They felt that the brochure provided an increased opportunity to educate clients, education that could potentially be translated into practice. The service providers reported that their overall involvement with the project itself was educational and that they appreciated the opportunity to meet as a group. It was also reported that reviewing the data from the RAI-HC was an “eye opener” for the service providers and one person spoke about its value in identifying issues to be addressed:

[I] look at that form. To me it’s an eye opener to make sure that it helped me to cover bases, to make sure I’m covering all the bases that need to be covered…I’d just like a tool that’s, as I said this at the beginning, that makes me think of things and makes sure I’m not forgetting something and there’s something in there that maybe would have slipped by. The RAI tool gives us a chance to catch that beforehand and work on it. ~ Service Provider 2

The project also opened the lines of communication between care coordinators and service providers and enabled all parties to come together.

[The] other half of that equation is that it opened up the door to communication. Now I can talk to the case manager. I had to talk to the case manager or the research assistant as the case may be about what was needed. But then I felt more comfortable and more accessible to talking about other things as well. So that was a positive that came out of [the research project]. ~ Service Provider 2

This was particularly important in light of the fact that communication had decreased significantly over time as various functions within the CCAC moved from relying on telephone conversations to a paper-based system.

### Theme 2: Improving the process

Care coordinators and service providers reported that there were areas for improvement which resulted in two sub-themes related to: a) the project itself; and b) their day-to-day practice.

### Areas for improvement related to the project

All participants felt that the project added to their workload and was further complicated by a lack of time, a lack of clarity or miscommunication stemming from various aspects of the research project. For the majority of the care coordinators and service providers, time was the most significant challenge.

I think mostly the challenges were around [pause] … it’s time consuming. [When] you have to get things organized to be sent to therapists, get packages all developed and sent to them. Also, what I found was that it’s the feedback: back and forth with the therapists. Depending on the therapist, there maybe was not that feedback from therapists and also with some of the therapists, we had to leave several voicemails to say “where is the information and can you please forward that to me?”. ~Care Coordinator 1

Several care coordinators also reported that it was difficult to know whether or not the intervention was working. It wasn’t clear to them during the project whether the therapists thought that sharing the RAI-HC data was useful or beneficial.

It was a lot of back and forth. I found it wasn’t really there (feedback) unless I was seeing the therapist, but yet there wouldn’t be time to discuss it specifically with them. Because we don’t often bump into the therapist in the community…And I don’t know if the therapists really found our information helpful. I never found that out. ~Care Coordinator 4

I’d really like to see before we go larger [in terms of research projects], to see if the therapist even thought it was beneficial because really we’re going to do the RAI no matter what so that’s not going to change our practice. But was the information beneficial to therapists because if it’s not beneficial to the therapists it really doesn’t matter because they still have to do their own assessments and I know that the RAI already encompasses some of their assessment tools anyways, so it would be more of whether it benefited them. ~Care Coordinator 2

One service provider also reported frustration with receiving very few referrals during the project. This was partially due to the particular numbers of clients that fit the study criteria and were identified and was also partially based on the current standard of practice that the CCAC uses in assigning new referrals for rehabilitation services that ensures equity across providers. Several therapists also spoke about the lag time between research participants and the continued need to be reminded about what they were supposed to do with respect to the intervention.

Service providers were also unclear as to why the project had included participants with cognitive deficits and questioned whether they had the ability to benefit from the project.

One of my issues was, I’d had several people that had cognitive deficits, so I found that a little difficult. I wasn’t quite sure why, whether they were good people to have, I mean that wasn’t my decision whether they were good to have in the study. You know, I was doing the Berg and the TUG on these people, but I didn’t think I was going to make a difference with their outcomes based on what the diagnosis was. So I was doing that and I wasn’t going to retest again because I wasn’t going to expect a difference and several cases I wasn’t going to be involved as far as therapy. I mean they identified they had a fall give them physio, but really because of the cognitive deficits they weren’t going to follow through and I had certainly 2 people, perhaps 3 like that, so that was a real issue. So you go ahead and you do the TUG and the Berg and you’re not going to see a change and you’re not going to retest this and you’re taking this person’s time to do this and I’m not sure, that felt uncomfortable. ~ Service Provider 5

I know it wasn’t about physiotherapy input being successful, that wasn’t what the study was about but I really struggle with these couple of people I had to do it on and I knew it wasn’t going to change and I had to take up their time and do all this. But I wasn’t going to retest it because I wasn’t going to see change or deal with the issue. So I found it, you know you go ahead and do all that testing, and okay why are we doing it? I was an unclear on that because it wasn’t to benefit the client because I wasn’t going, in either of these cases retest it because I wasn’t going to see change. ~ Service Provider 4

Lastly, service providers reported that they lacked a thorough understanding of the overall research project. They liked that they were included in the research, but would have appreciated more insight into the “whole picture” and a better understanding of the goals of the project.

### Areas for improvement related to daily practice

Participants also commented on areas for improvement that related to their daily practices of working in the community and working with one another. Care coordinators work closely with family members and service providers and make adjustments to the care plan based on their feedback. With a large number of clients on their caseload, it was challenging for one care coordinator to initiate follow-up with all of her clients. Data sharing between the care coordinators and the service providers could be one way to reduce workload and improve communication for service planning. Although sharing of the RAI data was felt to be useful, care coordinators and service providers discussed the impracticality of this given the current practices in place.

And in order to do that, you have to print off every RAI. And that’s a lot of paper for a case manager and the receiver, the person who’s receiving all that information, you can imagine being the community therapist and having [so many]visits a day, that would just be, all that paper work. ~Care Coordinator 3

We [complete the assessment] electronically and then we’d have to print it and its ends up being 12 pages of printed. What we were including in the study was 4 pages….but the full RAI is 12 pages and if you throw in the CAPs and all that, that’s a lot of pages you’re asking a therapist to read plus all the referral information and they have to do their own initial assessment. It adds a lot of time on to them and they’re not salary. ~Care Coordinator 2

Several suggestions were made as to how data sharing could be improved such as having an electronic form that could be accessed by multiple professionals and having some explanatory text to accompany the numeric scores from the RAI-HC (e.g., to describe the scores form the health index scales).

Duplication of assessments was also discussed as a problem with standard practice by both groups:

[The] way I see it and how it’s happening out there is that people are being assessed from so many different sources. Everyone that will go in their home, whether they’re therapists, there are a lot of other programs as well, case managers, everyone has their own set of questions and assessment to do. I think it would be important to see whether those, if by providing the information that we have from the RAI would cut down on the questions that are being asked to clients because clients are really getting fed up. So I’ve heard from therapists as well and it is, it’s our professional obligations and college that we have to do our own assessment to determine what is required. I really don’t know, I think we are still duplicating and duplicating a lot of questions that are being asked the same over and over to the same client from different sources. ~Care Coordinator 1

The other aspect is that as, as registered professionals, we need to be doing our own assessment anyways, so we can’t go by, okay the case manager says based on the RAI, the case managers telling us you know that this person has fallen here, but we still need to go in and look at, okay so they, I’m trying to think about what the RAI tells us. Like incontinence is an issue, well I’m still going to have to ask and confirm that, that it’s part of the issue or it might say they have incontinence as a falls risk. Well was their fall actually a result of rushing to the bathroom or slipping and, like I still have to look at all those aspects so, whether or not the RAI tells me that I’m still going to be investigating those factors. ~ Service Provider 4

Increased education about the RAI-HC was suggested as one avenue to assist in decreasing duplication and potentially reducing workloads. This sentiment was expressed by service providers who also highlighted the fact that a single education session was not adequate for some of the therapists.

I remember coming out of the instructor session thinking, kind of mental overload trying to understand everything they said and finding it useful. But then I knew as I walked out the door I was going to have to call someone and ask what do I do. Despite the fact that I’m sure I’m told what to do, but with the different forms I did feel some overload there. As to whether I can use the RAI in my practice, I’m assuming I’m using it in its components, but I don’t understand it enough to say I’m actually specifically using it as a tool in itself. ~ Service Provider 2

I think it comes back again to the RAI being a tool that is used as a universal tool. To say that this person gets a score of whatever and it doesn’t matter what that is. But I think it’s the same as the Berg for me, means something because I understand what the numbers mean. The RAI doesn’t do that for me because it’s not really something that I use in my everyday practice. So I think it comes down to again we still are always struggling to find tools that quantify or qualify what we do, one or the other. And I think because clinicians, especially those of us who’ve been practicing for a longer period of time, you’re accustomed to looking at what you do and saying, yes what I do is worth doing. ~ Service Provider 6

In summary, although the care coordinators and service providers reported that there were inefficiencies with the research project and with working together in the community, they believed that many of these issues could be remedied, primarily through increased education about the RAI-HC and its application to clinical practice.

## Discussion

This pilot project assessed the feasibility of additional education and data sharing between home care professionals as a means to better utilize the RAI-HC data in clinical decision-making. From the perspective of the care coordinators and service providers, the project led to an increased level of communication. This finding highlights the fact that even a relatively simple intervention can bridge an apparent gap between professionals. Other research in the home care sector has also highlighted the importance of interdisciplinary consultation and communication [13,30]. Home care professionals themselves have found that information sharing was instrumental in creating solutions to complex problems [13].

Although this project focused specifically on health care professionals working in home care, the importance of strengthening communication, through data sharing, has implications for health care providers working in other settings that provide care to complex seniors. In Ontario, long-term care homes, and complex continuing care facilities, also complete a version of the Resident Assessment Instrument and these findings can inform their communication strategies since multiple professionals (e.g., nurses, personal support workers, rehabilitation professionals) are involved in the planning and delivery of care to these individuals.

A new document, the falls checklist, was developed as part of this project and was used by both groups of professionals. The falls checklist was created based on feedback from the Clinical Advisory Group that they needed a simple one-page summary of the information provided in the falls Clinical Assessment Protocol (CAP) and based on earlier work that showed the importance of summary reports that are simple and highlight key pieces of information from the RAI-HC [9]. Although the focus group participants were not asked to comment on this tool specifically, there is the potential for something like this to be shared between care coordinators and service providers to improve communication and to quickly summarize their perspectives of the same client and highlight the key areas where they feel the client is at risk (e.g., environmental hazards, medications, unsteady gait).

It was evident from the focus groups that these participants feel increasing pressures on their time, which is supported from previous research in home care in Ontario [9,13]. This workload issue is not surprising given that there has been a dramatic increase in the number of individuals receiving home care in Canada within the last 10 years [31]. There is no simple solution to this situation, but it is worth noting that both groups of professionals cited this as one of the main issues they encountered in carrying out the project as well as a key barrier they face in daily practice. The participants also cited the use of the RAI-HC as a potential means to reduce duplication and streamline the assessment process. Although it could not be addressed in the current project, future research should more fully explore this finding to assess whether data sharing can lead to more efficient assessment in home care. Given that nearly 250,000 RAI-HC assessment are completed each year in Ontario, new ways to utilize these data can have important implications for the efficiency of the home care system.

An important objective of this project was to have a standardized method in place for sharing information from the RAI-HC between the CCAC care coordinators and the therapy providers. The focus groups highlighted how difficult it was for the service providers to use something new, like the RAI-HC, when they are not familiar with it and received minimal training. It was clear that our one-day education session was inadequate preparation and that ongoing follow-up was needed to ensure that all participants had a certain comfort level and understanding of how to interpret the information. Stolee et al. [9] also found that a key barrier to using health information in home care was inconsistent sharing of data from the RAI-HC between care coordinators and therapy providers and that the service providers did not have an adequate understanding of the RAI-HC and its outputs. If RAI-HC data are to be shared between professionals, there is a need for education and regular feedback and support as they become more familiar with the assessment. Finding ways to summarize the information that makes it meaningful to clinicians would improve its uptake [9].

At the time of writing, the service providers did not have electronic access to the RAI-HC data. It is possible to envision a time when all home care professionals will have access to an electronic record for each client. Little is currently known about how this type of system might lead to better client outcomes, however, communication between professional groups is vital [13] and has been cited as a means to improve the utility of these systems [32]. The availability of an electronic version of the RAI-HC, and its outputs, would address several of the key challenges cited in the focus groups, including printing and faxing large amounts of information and ensuring that the correct data are sent to the correct individual.

Ontario is currently involved in implementing a system that would address this issue, called the Integrated Assessment Record (IAR) [33]. This is an electronic application that allows authorized users to view a consenting client’s assessment information to effectively plan and deliver services to that client. The IAR allows assessment information to move with a client from one health service provider to another. Providers can use the IAR to collaborate with one another and to view timely assessment information electronically, securely and accurately. So although this is not fully operational, this is a major step in Ontario towards sharing of electronic health information, including the RAI-HC data.

A key limitation in this project was the fact these care coordinators and service providers came from a single geographic region of southwestern Ontario. However, given that this was a feasibility study, we were mainly interested in assessing whether this type of education and data sharing could work and learning about the types of challenges that arise when trying to implement something of this nature within normal clinical practice. We also have no reason to believe that the focus group participants were systematically different from home care professionals in other parts of Ontario. If anything, they may have been more motivated to address falls, and to use the study materials on a regular basis, since they volunteered for this project. This implies that if there was a bias, it would be towards over-estimating the success of this project.

The project was based on the assumption that inter-professional communication and collaboration is related to improved client outcomes. Unfortunately, we are unable to report whether this process of enhanced education and data sharing led to improvements in client outcomes. Among the clients who had a new fall and were eligible to receive in-home rehabilitation during the study timeframe, our sample size was very small (n = 34) and we did not have adequate statistical power to determine whether these clients had outcomes that differed from the usual care group. This would be important to address in future research in order to determine to what extent improved data sharing can influence both the process of care and outcomes of care, which are key domains to measure in assessing the quality of care provided.

## Conclusion

This project facilitated improved communication between home care professionals but there still exist a number of challenges to successful data sharing and uptake of information from the RAI-HC. Some of these challenges could be addressed through more intensive education and by making the information available to all professionals in an electronic format that is easy to interpret. Future research of a longitudinal nature could shed light on whether enhanced provider education and information sharing can lead to improved client outcomes. Our results suggest that if certain barriers are addressed, there is the potential for sharing certain pieces of the RAI-HC data between home care professionals which could ultimately lead to improved communication and enhanced quality of care.

## Abbreviations

CAP: Clinical assessment protocol; CCAC: Community care access centre; RAI-HC: Resident assessment instrument for home care.

## Competing interests

The authors declare that they have no competing interests.

## Authors’ contributions

DG conceived of the project, was the study’s Principal Investigator, oversaw all aspects of the study including data collection and analysis and took the lead in drafting the manuscript. RP was the study’s research coordinator, was involved in all aspects of the study and reviewed the manuscript. PF took the lead in the analysis of the qualitative data and reviewed the manuscript. HJE was the key representative from the Waterloo-Wellington CCAC, was highly involved in coordinating data collection and reviewed the manuscript. The other co-authors (JPH, PS, JP, AP, KB) were part of the research team, provided feedback throughout the project regarding data collection and analysis and reviewed the manuscript. All authors read and approved the final manuscript.

## Pre-publication history

The pre-publication history for this paper can be accessed here:

http://www.biomedcentral.com/1471-2318/14/81/prepub
